# Achieving the Sustained Virologic Response With a Short-Course Treatment of Sofosbuvir/Velpatasvir

**DOI:** 10.7759/cureus.37126

**Published:** 2023-04-04

**Authors:** Simon P Abi-Saleh, Fatima Ghazal, Eva Urtasun Sotil

**Affiliations:** 1 Internal Medicine, University of Connecticut School of Medicine, Farmington, USA; 2 Gastroenterology and Hepatology, Hartford Hospital, Hartford, USA

**Keywords:** hepatitis c management, decompensated liver cirrhosis, sofosbuvir/velpatasvir, sustained virologic response, hepatitis c (hcv) infection

## Abstract

Hepatitis C, a single-stranded RNA virus officially discovered in 1989, is one of the most known viruses of the *Flaviviridae* family. Direct-acting antiviral drugs helped revolutionize the management of hepatitis C infection by guaranteeing higher cure rates. The medical field has strived to optimize the management of this disease, with recent reports proposing a shorter treatment duration to achieve the sustained virologic response (SVR). We present a case of a patient diagnosed with hepatitis C decompensated liver cirrhosis who achieved the SVR after only two weeks of treatment with sofosbuvir/velpatasvir, suggesting that short-term therapy might be beneficial for these patients.

## Introduction

Hepatitis C infection is a highly prevalent disease that was first described in the 1970s as a non-hepatitis A or B virus [1]. New therapies are continuously generated in order to optimize treatment and improve public health. Recently, some reports have suggested that patients can achieve the sustained virologic response (SVR), defined as an undetectable amount of HCV RNA in the serum for at least three weeks after completion of treatment, with a shorter duration of therapy [2-4]. We present a case of a patient with hepatitis C decompensated liver cirrhosis who achieved the SVR with only two weeks of sofosbuvir/velpatasvir, further suggesting that short-term therapy might be beneficial for these patients.

## Case presentation

A 61-year-old female was being followed at the hepatology clinic for the management of hepatitis C-induced liver cirrhosis. She carried a past medical history significant for essential hypertension, cerebral aneurysm, cerebrovascular accidents, subarachnoid hemorrhage, iron deficiency anemia, and diverticulitis. The patient contracted the virus after receiving blood transfusions during her first pregnancy. She was previously initiated on pegylated interferon therapy, but this treatment was eventually discontinued as she did not respond to therapy. Years later, she established care at the hepatology clinic. She had already developed decompensated liver cirrhosis with multiple complications that included ascites, non-bleeding esophageal varices, and hepatic encephalopathy. She had evidence of active hepatitis C infection with a viral load of 15098 IU/mL, genotype 1a.

The patient had indications for a liver transplant and was amenable to treatment. However, she was deemed not to be a transplant candidate because of insufficient social support. The patient developed further complications of liver cirrhosis including hepatocellular carcinoma (HCC) complicated by rupture of the liver lesion. Therefore, treatment for hepatitis C was initiated with sofosbuvir and velpatasvir (Epclusa). Two weeks after initiating treatment, the patient developed a full-body rash that was believed to be an allergic reaction secondary to Epclusa. The rash subsided after the medication was held, and the patient was treated with antihistamines. Hepatitis C viral load was tested after that episode, and the viral RNA was undetected. This test was repeated at months 4, 8, and 13 with the viral load remaining undetected. The patient had achieved the SVR with only two weeks of treatment.

## Discussion

Hepatitis C is an exceptionally prevalent disease with more than 185 million cases worldwide [5]. The virus genotype differs between regions, but genotype 1 is estimated to be the most common at 46.2% [6]. Hepatitis C was originally found to be responsive to interferon-based therapy, notably interferon alpha; however, the SVR was low with rates ranging from 6% to 20%. In an attempt to improve the SVR, pegylated interferon and ribavirin were added to treatment. The major breakthrough in the management of hepatitis C was the introduction of direct-acting antiviral drugs (DAAs) that targeted proteins involved in the viral replication cycle. Since their authorization by the FDA in 2011, this new therapeutic regimen tremendously improved the SVR [7]. In 2015, the ASTRAL-1 trial introduced the fixed-dose combination of sofosbuvir and velpatasvir for the treatment of all HCV genotypes, except genotype 3, for a 12-week period. Sofosbuvir is a nucleotide inhibitor of the NS5B polymerase protein, and velpatasvir is an NS5A polymerase inhibitor, both of which are essential components of the HCV replication cycle. This double-blinded, placebo-controlled trial included patients with or without compensated cirrhosis who were either treatment naïve or treated with interferon-based therapy but had not achieved the SVR; 99 % of the 624 patients achieved the SVR after 12 weeks of treatment. Moreover, 98% of 210 patients with genotype 1a successfully eradicated the virus [8]. In 2020, Mangia et al. performed a pooled global analysis of 12 clinical cohorts which included patients with all six HCV genotypes with or without compensated cirrhosis treated with Epclusa [9]. Out of the 5552 patients, the SVR was achieved in 98.9% in patients with all genotypes and 99.1 % in patients with genotype 1. These impressive findings demonstrated that Epclusa can be used for the treatment of HCV without predetermining the viral genotype. Furthermore, sofosbuvir/velpatasvir was efficient in treating HCV in patients with decompensated liver cirrhosis as it achieved 88% SVR in genotype 1 [10]. Historically, studies on patients with HCV genotype 1 showed that the treatment duration with DAAs ideally required 12 weeks to achieve the SVR. Our case was unique as the patient achieved the SVR with only two weeks of treatment with Epclusa. Investigations for short-duration therapy for hepatitis C have been explored in the past. For instance, an open-label study enrolled 27 patients with HCV type 1 and 4 with concomitant chronic HIV and proposed an eight-week treatment with ledipasvir/sofosbuvir. On week 12, the SVR was achieved in all participants of this small cohort [11]. Other studies have also shown efficacy in treating HCV with short-duration DAA therapy, but these studies were based on a relatively small number of participants [2-4]. A six-week treatment with Epclusa was studied in the REACT trial, a randomized open-label study, that enrolled 188 patients with HCV. The study was terminated early because of the high virologic relapse rate, and a six-week treatment was not found to be non-inferior to 12 weeks [12]. It is important to note that the study included a majority of male patients with evidence of HCV RNA within six months of enrollment, and it did not include patients with decompensated liver cirrhosis and HCC. In our case, the patient was a female with decompensated liver cirrhosis, HCC, and evidence of hepatitis C infection for several years. Eradication of hepatitis C was sustained for at least one year after short-term therapy. Therefore, it would be interesting to study this subset of the HCV population and assess the benefit of a shorter treatment duration with sofosbuvir/velpatasvir. While this is only an isolated case, a shorter duration of treatment would not only benefit patients from an economic standpoint but can also decrease the rate of non-adherence to this prolonged treatment. It is also possible that the patient might have undergone spontaneous viral clearance facilitated by the inflammatory response in the setting of her allergic reaction.

## Conclusions

The management of hepatitis C infection has been rapidly evolving in the past decades. DAAs have positively impacted the survival of this population subset by almost guaranteeing the SVR. Multiple studies have challenged the treatment duration of HCC with DAAs, opting for a shorter therapy duration. Sofosbuvir/velpatasvir is one of those potential agents that might show great treatment efficacy with a treatment period shorter than 12 weeks.
